# Effect of Fortification with Multiple Micronutrient Powder on the Prevention and Treatment of Iron Deficiency and Anaemia in Brazilian Children: A Randomized Clinical Trial

**DOI:** 10.3390/nu13072160

**Published:** 2021-06-23

**Authors:** Malaine Morais Alves Machado, Mirella de Paiva Lopes, Raquel Machado Schincaglia, Paulo Sérgio Sucasas da Costa, Alexandre Siqueira Guedes Coelho, Maria Claret Costa Monteiro Hadler

**Affiliations:** 1 Graduate Program in Health Sciences of the Federal University of Goiás, Faculty of Medicine, Federal University of Goiás, Goiânia 74605-050, Goiás, Brazil; malainenut@ufg.br; 2 Faculty of Nutrition, Federal University of Goiás, Goiânia 74605-050, Goiás, Brazil; mirelladepaivalopes@gmail.com; 3 Research Laboratory in Clinical and Sports Nutrition, Faculty of Nutrition, Federal University of Goiás, Goiânia 74605-050, Goiás, Brazil; raquelms@outook.com; 4 Department of Pediatrics, School of Medicine, Federal University of Goiás, Goiânia 74605-050, Goiás, Brazil; paulosucasas@ufg.br; 5 Graduate Program in Nutrition and Health of the Federal University of Goiás, Faculty of Nutrition Goiânia, Federal University of Goiás, Goiânia 74605-050, Goiás, Brazil; alexandre_coelho@ufg.br

**Keywords:** iron deficiency, anaemia, micronutrients, deficiency diseases, children

## Abstract

Fortification with multiple micronutrient powder has been proposed as a public health intervention able to reduce micronutrient deficiencies in children. Our objective was to compare the effectiveness of fortification with multiple micronutrient powder with drug supplementation in the prevention and treatment of iron deficiency and anaemia. This was a cluster trial with anemic and non-anaemic children between six and 42 months old, in randomization data. Non anaemic children received fortification with multiple micronutrient powder or standard drug supplementation of ferrous sulfate associated with folic acid in a prevention dose. Anaemic children who were randomized to receive multiple micronutrient powder also received the recommended iron complementation for anaemia treatment. A total of 162 children were evaluated. The prevalence of anaemia decreased from 13.58 to 1.85%. Iron deficiency decreased from 21.74% to 7.89% (by serum ferritin) and iron deficiency decreased from 66.81 to 38.27% (by soluble transferrin receptor). No difference was identified between interventions for hemoglobin (*p* = 0.142), serum ferritin (*p* = 0.288), and soluble transferrin receptor (*p* = 0.156). Fortification with multiple micronutrient powder was effective in preventing iron deficiency and anaemia in children aged six to 48 months. In anaemic children; it was necessary to supplement the dose of multiple micronutrient powder with ferrous sulfate.

## 1. Introduction

Anaemia remains a global public health problem, especially in countries in Africa and South Asia [1,2,3]. The most prevalent type is iron deficiency anaemia (ID), and the most affected population groups are women of reproductive age and children <5 years. The worldwide prevalence of anaemia in children was estimated at 43% [1] (600 million) [2]. In Brazil, the mean anaemia in those <5 years old ranged from 36.4% [8.41–51.07] in 1990 to 25.07% [11.33–72.22] in 2016 [3].

In Latin American countries such as Brazil, anaemia persists [3], especially because other nutritional deficiencies that affect anaemia rates still coexist [4]. One of the six global goals of the 2025 World Health Assembly Global Targets to improve maternal and child nutrition was to reduce the prevalence of anaemia by 50% [5]. In this sense, some interventions in the world have been proposed to reduce this disease, among them, fortification with multiple micronutrients powder (MNP) [4,6,7,8]. This intervention was initiated in Brazil in 2015, aiming to correct anaemia and other nutritional deficiencies [4].

This is the first study with Brazilian children and one of the first in the world to compare fortification with MNP to supplementation with ferrous sulfate [9,10,11] and folic acid. In general, the existing studies have compared fortification with MNP to no intervention, or a placebo [12,13,14,15,16,17,18] or other interventions such as dietary guidance [19] and parental education [20]. In addition, this study evaluated a wider age group than most studies and provided information on adverse reactions (AR) to the use of MNP, which are scarce in the literature [7].

A clinical trial with cluster randomization was conducted to verify the effectiveness of MNP in the prevention and treatment of ID and anaemia in children aged six to 48 months, when compared to supplementation with ferrous sulfate and folic acid (FS_FA). The hypothesis was that the MNP sachet is able to prevent ID and anaemia in children and treat anaemia when associated with iron.

## 2. Materials and Methods

### 2.1. Study Design and Eligibility

This was a randomized clustered clinical trial, open in parallel, lasting 15 (14–16) weeks and supplying a target of 60 sachets/child [21]. This study was part of the matrix research “Effectiveness of fortification with powder micronutrients in prevention and treatment of micronutrient deficiency: a randomized clinical trial”, from the Faculty of Nutrition of the Federal University of Goiás, Goiás, Goiânia, Brazil.

The sample consisted of children from Early Childhood Education Centers (CMEIs) in the city of Goiânia, Goiás, located in the Midwest of Brazil. Early childhood education comprises the first stage of basic education. In Brazil, it ranges from six months to five years and 11 months. The CMEIs are public institutions, that is, they are totally free and linked to the Municipal Department of Education. The professionals of the CMEIs are teachers graduated in pedagogy, who take care of the children for up to eight hours a day when their parents are working. Therefore, an important part of the care with hygiene, feeding and development of children is offered locally, and becomes a relevant scenario for fortification with MNP. These institutions are supported by the Health at School Program (PSE), through which public health teams work within CMEIs with health prevention and promotion activities, such as food and nutrition education. Children between 6- and 42-months old were eligible at the date of recruitment, as they would be 48 months old at the end of the intervention. Premature, twin, and low birth-weight children, as well as children undergoing treatment for anaemia, malaria, HIV, or hemoglobinopathies, and those who were allergic to any components of the intervention were excluded.

### 2.2. Sampling and Randomization

The CMEIs of the city that already received MNP, those without a nursery, or that were not working full-time were excluded. Next, two CMEIs were drawn per health district (HD), and later one CMEI of each HD was chosen to receive MNP and the pair received FS_FA. This draw was conducted through a list of random numbers (Epi info 6.04d^®^, Centers for Disease Control and Prevention). Randomization was stratified by gender and age group by means of a simple random draw in Excel^®^ (2010).

Sample calculation a priori was performed considering a clinical trial study, in parallel, two-tailed type, absolute error of 5%, test power of 80%, allocation rate of 1:1, and an effect size of 0.38, based on hemoglobin [22]. To cover losses, the sample (*n* = 164) was increased to 22%, totaling 200 children (GPower 3.1.9.2^®^).

### 2.3. Intervention

From the hemoglobin concentration at the baseline, the children were randomized to both interventions and classified as anaemic or non-anaemic. These were subdivided into: Group A: Anaemic—MNP (60 sachets) + 3.2 mg/Kg/day of elemental iron (EI) (Anemifer^®^ gotas—drops—Pharmascience Laboratórios Ltd.a., Betim, MG, Brazil—125 mg of heptahydrate ferrous sulfate per mL and 25 mg of elemental iron per mL); Group B: Non-anaemic—MNP (60 sachets); Group C: Anaemic—4.2 mg/kg/day of EI (Anemifer^®^ gotas—drops—Pharmascience Laboratórios Ltd.a., Betim, MG, Brazil); + 50 μg folic acid (FA); Group D: Non-anaemic—EI (1.4 mg/kg/day) + 50 μg FA. In groups C and D, treatment and prophylaxis were adopted, according to Hadler et al. [23].

The median and interquartile range of elemental iron given to anaemic children for the 6–12 months, 12–24 months, 24–36 months and 36–42 months were 38.64 (28.32–41.79), 39.20 (28.20 48–43.68), 41 (35.28–51.56) and 47.63 (24.92–70.35) mg/day, respectively. These amounts are equivalent to 31 (23–33), 31.5 (23–35), 32.5 (28–41) and 38 (20–56) drops of heptaidratated ferrous sulfate (Anemifer^®^). In non-anaemic children, a median dose of elemental iron and interquartile intervals for these same age groups were 11.62 (11.34–14.18), 15.19 (1 3.3–16.1), 18.66 (17.36–20.33) and 21.49 (19.46–23.66) mg/day. These quantities for the same ranges are equivalent to 9 (9–11), 12 (11–13), 15 (14–16) and 17 (16–17) drops of heptaidratated ferrous sulfate (Anemifer^®^).

The sachets were manufactured by DSM Nutritional Products Colombia S.A^®^, Cundinamarca, Columbia imported and supplied by the Brazilian Ministry of Health. The sachet contains 15 micronutrients (sachet 1 g = 400 μg retinol acetate, 5 μg colecaciferol, 5 mg alpha-tocopherol acetate, 30 mg ascorbic acid, 0.5 mg thiamine nitrate, riboflavin and pyridoxine hydrochloride, 6 mg niacinamide, 0.9 μg cyanocobalamin, 150 μg folic acid, 10 mg encapsulated ferrous fumarate, 4.1 mg zinc gluconate, 560 μg copper gluconate 17 μg sodium selenium and 90 μg potassium iodide) [24]. FS was the Anemifer^®^ oral solution (25 mg elemental iron/mL—PharmaScience, Minas Gerais, Brazil) and FA was the Folacin^®^ oral suspension (0.2 mg/mL—Arese Pharma, Valinhos, Brazil).

Parents and the institution were instructed to maintain the children’s usual dietary pattern. The interventions were administered by the teachers, who were trained, and occurred in the CMEIs. The researchers delivered doses sufficient for 30 days of intervention, which were stored in the CMEIs themselves, protected from light, heat and humidity, and after this period, the remainder were delivered. Every week the researchers followed the administration in the CMEIs to check if the established protocol was being followed and counted the empty sachets for verification. The number of vials of FS and FA per child was calculated based on the days expected to last each vial.

The MNP sachet was mixed in a small portion directly on the child’s plate, rice or vegetable puree, depending on the CMEI menu each day. The meal that was received the intervention was always lunch, Monday to Friday. The intervention FS_FA was also administered by the teachers, in the CMEIs themselves, from Monday to Friday 1 h before lunch. In this case, the number of drops equivalent to the child’s dose was described on a label glued to the child’s bottle and the teacher counted the drops and administered them with the aid of a spoon.

The teachers recorded the daily doses administered and the occurrence of AR, through a control spreadsheet was elaborated by the researchers and left with the teachers. This worksheet was individualized to the child. For absentee children, a telephone follow-up was carried out with guardians who administered these doses at home. In this case, for MNP intervention, the sachet was added in part of rice or vegetable puree at lunch and for the intervention FS_FA, the drops were counted and administered 1 h before lunch, with the aid of a spoon.

### 2.4. Variables Recorded

The child’s age (date of birth), gender (male/female), maternal age (years of life), parents’ schooling (full years), skin color (self-reported by guardians), per capita family income and economic class [25] were recorded. Additionally, hemoglobin (anaemia < 11 g/dL) [26], C-reactive protein (CRP) (presence of inflammation > 0.5 mg/dL) [27,28], alpha-1 acid glycoprotein (AGA) (presence of inflammation > 100 mg/dL) [27,28], serum ferritin (ID in the presence of inflammation: <30 ng/mL and in the absence of inflammation <12 ng/mL) [28], and transferrin soluble receptor (sTfR; ID > 1.76 mg/L; value attained from the analysis kit) were determined.

### 2.5. Data Collection

Collection took place between March 2018 and March 2019. The interviews were conducted with those responsible for nutrition and trained nutrition students. Blood collection was performed by a technician from the laboratory who performed the analyses. Children who did not attend CMEI on the blood collection date had this collection performed at home or in the laboratory. Venous blood was collected after fasting for 3 (<12 months) to 8 h.

The blood count was analyzed by electronic count (XE-2100—Sysmex—Roche^®^, Indianapolis, IN, USA), CRP by turbidimetry (Cobas 8000 c502—Roche^®^, Mannheim, Germany), and AGA by immunoturbidimetry (AU—Beckman Coulter^®^, Brea, CA, USA) The random error (CV%) for blood count and AGA was <10% and for CRP was <5%.

Serum ferritin and sTfR were dosed in serum by chemiluminescence (DXI-800—Beckman Coulter^®^, Brea, CA, USA) and nephelometry (BN II—Siemens^®^, Berlin, Germany), respectively. The random error (CV%) for serum ferritin was <8% and for sTfR was <7.9%.

### 2.6. Statistical Analyses

The data were processed using double entry (Epi Info^®^ 6.04d). The significance level for all tests was 5%.

Descriptive analysis was performed, which for continuous data were presented as the mean and standard deviation. Data normality was tested by the Shapiro–Wilk test. For categorical variables, the data were presented in absolute (*n*) and relative (%) values. Pearson’s Chi-Square test or Fisher’s Exact test was performed to compare the proportions between groups, and the Student *t* or Mann–Whitney U tests were used for continuous variables. Stata 16 software was used in this analysis.

The prevalences were obtained by logistic regression, correcting for the effects of age and number of doses for each intervention. To evaluate the effect of the intervention on the main (hemoglobin) and secondary (ID) outcomes, mixed linear models were used, assuming the design with split plots, in which the CMEIs were considered as sub-plots of the HD. In cases where these effects were not detected as significant, they were removed from the model. The analyses were conducted considering age as a covariate at the baseline and after the intervention. The number of doses was also considered as a covariate in the data analysis after the intervention. The variables CRP and AGA were considered covariates in serum ferritin analyses.

The normality of the residues of each model was evaluated graphically and through the Shapiro–Wilk test. Despite the significance of the deviations of normality observed in serum ferritin and sTfR analyses at the baseline, we chose to use the parametric approach for all outcomes because the results were completely consistent between approaches (parametric and non-parametric). The effect size was calculated using the Cohen d-test. Moreover, in the parametric approach, it was possible to correct for the effect of the covariates in each model. All analyses were performed using the intention to treat in software R v. 4.0.3.

### 2.7. Ethical Considerations

This study was approved by the Research Ethics Committee of the Federal University of Goiás (protocol 3,692,768). The study was recorded in the Brazilian Clinical Trial Records—REBEC (Protocol RBR-4hm7mz).

## 3. Results

### 3.1. Participants

Sample characterization data are described in Table 1.

**Figure 1:**
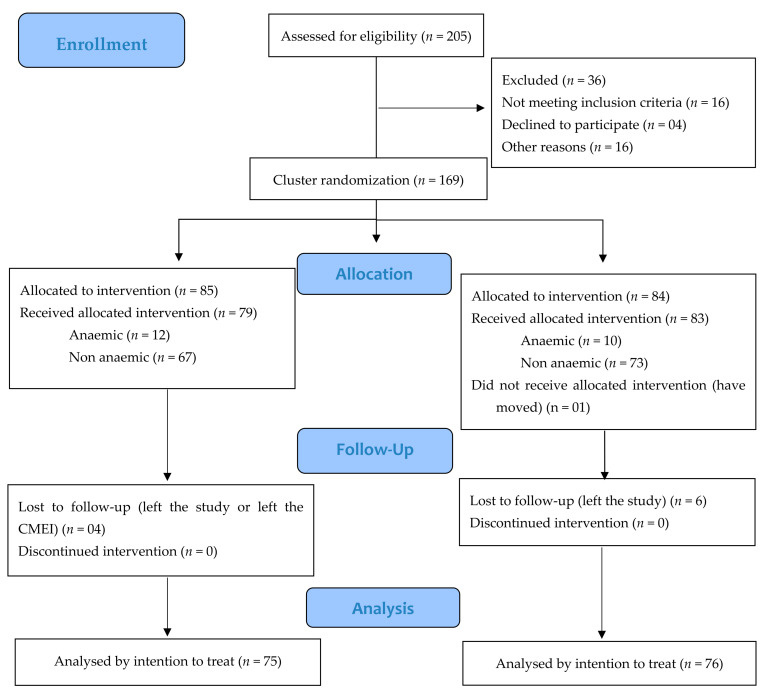
Profile of this randomized clinical trial. The Early Childhood Education Center (CMEI) that received allocation for multiple micronutrients powder (MNP) in a health district had its peer in the same health district receiving ferrous sulphate and folic acid (FS_FA), defined by lot, totaling 10 CMEIs (5 MNP and 5 FS_FA). Within each of these CMEIs, if children were anaemic, they received the dose corresponding to the anaemia treatment recommendation, and if they were not anaemic, they received the dose corresponding to the prevention of anaemia. The number of children included in the intention-to-treat analysis corresponded to those for whom initial and final hemoglobin levels were measured.

Variables did not differ from the baseline. Of the 205 children evaluated for eligibility, 169 were chosen and randomized (Figure 1), and 162 children received the intervention. The median number of treatment doses was 46 (40–53) for MNP and FS_FA groups. The median and interquartile ranges (IR) for weight among anaemic children at baseline was 10.8 kg (IR: 9.95–12.65 kg) and among non-anaemic children 13.40 kg (IR: 11.65–15.65 kg).

### 3.2. Prevalence of Anaemia and Iron Deficiency

The overall prevalence of anaemia at the baseline was 13.58% and decreased to 1.85%. The overall ID evaluated by serum ferritin, which was adjusted for inflammation, went from 21.74 to 7.89%, and the overall ID evaluated by sTfR decreased from 66.81 to 38.27%.

In children <24 months, the prevalence of anaemia went from 26.03 to 2.74% and ID from 26.39 to 7.35% when serum ferritin was evaluated. Prevalence decreased from 75.34 to 45.21% when sTfR was evaluated. The prevalence of anaemia and ID are described in Table 2 by the type of intervention.

### 3.3. Random Effects

The existence of intraclass autocorrelation was not observed when the random effect of HD or CMEI was evaluated for any of the outcomes analyzed at the baseline or after the intervention, i.e., children belonging to the same HD or CMEI did not interfere in the analysis. Therefore, we opted for a fixed effect model in these analyses.

### 3.4. Effect of Baseline Intervention

Age had a significant effect on hemoglobin (*p* < 0.001) and sTfR (*p* < 0.001) but not serum ferritin (*p* = 0.359). For hemoglobin (*p* = 0.909), serum ferritin (*p* = 0.378), and sTfR (*p* = 0.294), there was no difference between interventions, but there was a difference between groups (anaemic and non-anaemic) within the interventions for hemoglobin (*p* < 0.001) and sTfR (*p* < 0.001) (Table 3).

**Figure 2:**
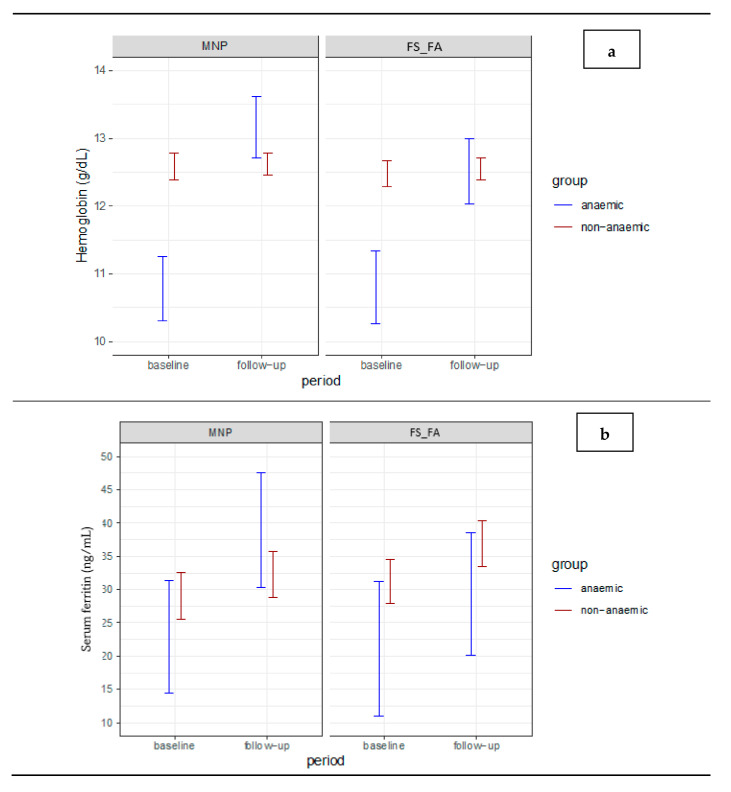

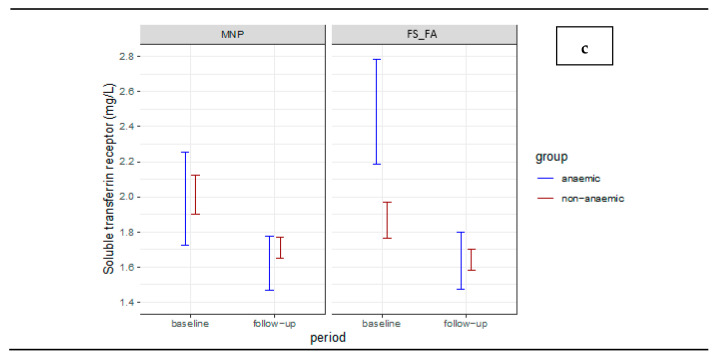
Average estimates of hemoglobin (**a**), serum ferritin (**b**), and soluble transferrin receptor (**c**) levels for each group and intervention in the two evaluation periods. Estimates of means with non-overlapping error bars differ statistically from each other, at a 5% level of significance.

In this model, age at the baseline, effect of the individual, and number of doses received were considered as covariates. Age influenced hemoglobin (*p* < 0.001) and sTfR (*p* < 0.001), but not serum ferritin (*p* = 0.283). The effect of the individual was significant for the three outcomes evaluated (*p* < 0.001), and the number of doses was not significant for any of the outcomes (*p* > 0.05). The means for hemoglobin, serum ferritin and sTfR are represented in Figure 2.

### 3.5. Delta Analyses

For hemoglobin, intraclass autocorrelation was demonstrated within the CMEIs, in 12.4% of the total residual hemoglobin variation, which was due to a variation between the CMEIs (*p* = 0.011). In this case, we opted for a complete mixed model, which included the effect of CMEI.

For serum ferritin and sTfR, the existence of a random effect of HD or CMEI was not evidenced, and these two outcomes were used for a fixed effects model. There were no differences between the interventions for any of the outcomes (Table 4).

### 3.6. Adverse Reactions

The most frequent adverse reaction (AR) of MNP and FS_FA was softened stool/diarrhea, which occurred in 10.26% (*n* = 8) and 15.66% (*n* = 13) of children, respectively (*p* = 0.309). There were no reports of vomiting. When evaluating nausea, intolerance, darkened feces, constipation, fever, and respiratory infections, the frequencies were less than 10%, with no difference between the groups. No child had intervention suspended due to reported AR.

## 4. Discussion

This randomized clinical trial demonstrated that the use of MNP was as effective as the use of FS_FA to improve serum levels of hemoglobin, serum ferritin, and sTfR, i.e., there was no difference between the two interventions. Additionally, the difference between the groups within the same intervention was due to the difference in the initial prevalence of anaemic and non-anaemic children in the sample. In the case of anaemic children, when a treatment dose was used, anaemia almost reached 1%.

Fortification with MNP is one of the public health strategies used in various parts of the world to reduce ID and anaemia among children [5,7]. In Brazil, the prevalence of anaemia has decreased [3], and our findings follow this trend. However, as the scope of fortification with MNP still does not reach all early childhood education institutions in every city, this may also be due to other efforts, such as flour fortification [29], greater assistance to pregnant and lactating women in primary care [30,31], preventative supplementation with iron [32] and vitamin A [33], stimulation of exclusive breastfeeding [34], and stimulation of umbilical cord clamping in a timely manner [35].

The studies analyzed by De-Regil et al. [8] in countries in Africa, Asia, and Haiti used 5 to 15 micronutrients in the sachets, and all contained iron, zinc, folic acid, vitamin A, and vitamin C in their composition. This study showed that children aged six to 23 months who received MNP had a reduced risk of anaemia (by 31%) and ID (by 54%) after 2 months or more of intervention, when compared with no intervention or the placebo, regardless of the initial prevalence of anaemia [5].

In a more recent review, with children aged 2 to 12 years in Latin America, Africa, and Asia, the use of MNP compared to a placebo reduced anaemia by 34% and DF by 35% [2]. For ethical reasons, in our study, a placebo was not used.

The amount of iron contained in the sachet used in our study (10 mg) was lower than other studies [9,10,13,36,37,38]. However, the prevalence of anaemia in the population we evaluated was low, and there was a positive effect on ID and anaemia. In addition, the sachet contained other vitamins and minerals to improve the nutritional quality of food, which is also the focus of the Brazilian intervention [22].

The sachet avoids the error of inexact counting of drops and dispenses a fraction, which reduces the potential for overdose. It is simple to store, transport, and distribute and does not darken one’s teeth or cause gastric irritation [4]. In addition, it maintained the child’s usual feeding practices, facilitating the transition from breastfeeding to food introduction. It is, therefore, an effective and viable alternative to conventional supplementation with FS. In those children who manifested food refusal, supplementation with FS was a more viable alternative because it is independent of food acceptance.

We identified lower than expected intervention support, with no difference between the groups (*p* = 0.932). Of the sample, 9.88% received 60 doses, 29.01% received 50–60 doses, 56.17% received 36–50 doses, and 4.94% received less than 36 doses, even with training and monitoring of the researchers. Another study reached 39% of adherence [14]; however, it was an intervention that lasted half the time as that proposed in the present study.

In our study, the children received an average of 3.24 ± 0.07 sachets/week. In some studies, the number of subjects ranged from 32 to 90%, considering high-strength consumption of 4 sachets/week or more [13,36,39,40]. The highest fit was observed in trials in which the children received the product intermittently [39,41]. This regimen could reduce mental pressure and anxiety about the daily supply of the supplement to children.

A study of 389 anaemic children aged 6 to 59 months in Tanzania showed that children who received 3–5 sachets per week had a greater reduction in anaemia when compared to those who received 1 sachet per week [41]. However, in this study the fortification was performed at home and not in an institution.

The weekly offer was lower than expected (5 times/week) in our study due to the irregular attendance of children in the CMEIs. This was due to reported colds, trips, holidays, strikes/outages, and teacher meetings in the CMEIs. In a CMEI, we recorded an episode of a hand-foot-mouth syndrome outbreak, which resulted in the transient interruption of the intervention at the site. However, this demonstrates how the reality of MNP supply can be done in institutions. One possibility to improve adherence to CMEIs may be that, in the case of calendars with a reduced number of school days or school leave, parents may offer complementary doses at home.

The clinical significance of anaemia in children is an indisputable issue. However, at a recent WHO meeting [41], defining the effect of differences in hemoglobin concentrations on cognitive and physical development in children is still an open question. Carter et al. [42] observed a small cognitive and recognition memory impact in babies with hemoglobin <10.5 mg/dL compared to >11.0 mg/dL. In this context, probably some differences of 0.1 mg/dL observed in our study will not have an impact on the clinical outcome, despite presenting statistical significance, but this issue needs further studies to be clarified.

The ARs to the use of FS are known, but there are few studies with AR data on the use of MNPs [1]. In this study, the frequency of AR was low; therefore, the use of MNP by children in this age group was safe. However, there is already evidence that the use of MNP sachets containing iron can negatively alter the microbiota of children [43], which could be the subject of future investigations in children receiving MNP.

Among the strengths of this study we can highlight: (1) its pioneering quality because it is the first national study with this design that evaluated the efficacy of MNP in the prevention and treatment of anaemia; (2) it has responded to the needs of a public policy in a country that already has a high prevalence of ID due to anaemia; (3) the evaluation compared two interventions and not a placebo; and (4) it was a randomized study, with a cluster design that was representative of different social and demographic strata of the population.

One limitation of the study was the absence of blinding because the object of study (powder) and pattern (liquid) presented different forms of presentation. However, there was blinding of the evaluators of the tests to minimize the possible effects of this variation. To avoid errors in the administration of the supplement, a CMEI intervention was used, so that for each pair of CMEIs in the same HD, one received MNP and the other FS_FA.

The intake of dietary iron was not investigated in the present study. However, all children received the same menu calculated and standardized by a nutritionist of the National School Feeding Program during the period they were at CMEI (breakfast, morning snack, lunch, afternoon snack and dinner).

## 5. Conclusions

Fortification with MNP was effective in preventing ID and anaemia in children aged six to 48 months. In anaemic children, it was necessary to supplement the dose of MNP with FS. These results suggested that children who receive iron interventions require a prior biochemical evaluation to verify the adequacy of the dose used (prevention or treatment of ID and/or anaemia). For children who are absent from the CMEIs, complementation of doses at home may be an alternative to improve the use of MNPs.

## Figures and Tables

**Table 1:** Characterization of the sample at the baseline (*n* = 162).

Variables ^1^	Total (*n* = 162)	MNP ^2^ (*n* = 79)	FS_FA ^3^ (*n* = 83)	*p* Value ^4^
Gender (female %)	83 (51.23)	41 (49.40)	42 (50.60)	0.869
Child’s age (months)	24 (14–35)	24 (16–35)	24 (13–35)	0.975
<24 months	73 (45.06)	36 (49.32)	37 (50.68)	0.899
≥24 months	89 (54.94)	43 (48.31)	46 (51.69)	
Child skin color *n* (%)				0.598
White	73 (45.06)	39 (53.42)	34 (46.58)	
Brown	80 (49.38)	36 (45.00)	44 (55.00)	
Black	9 (5.56)	4 (44.44)	5 (55.56)	
Day care time (months)	14 (4–24)	15 (4–24)	14 (4–24)	0.584
Maternal age (years)	29.99 ± 5.96	30.44 ± 6.03	29.56 ± 5.88	0.349
Mother’s schooling (complete years)	12 (12–15)	12 (12–15)	12 (12–16)	0.826
Father’s schooling (complete years)	12 (10–12)	12 (10–12)	12 (10–13)	0.818
Economic class ^5^				0.912
A or B	54 (33.33)	26 (48.15)	28 (51.85)	
C, D, or E	108 (66.67)	53 (49.07)	55 (50.93)	
Per capita income (Real ^6^)	750 (500–1000)	670.83 (500–1000)	750 (450–1000)	0.893

^1^ Variables with normal distribution are presented as mean ± SD, and variables without normal distribution are presented as median and interquartile range. ^2^ MNP = Multiple micronutrients powder. ^3^ FS_FA = Ferrous sulfate and folic acid. ^4^ Pearson’s chi-square test/Fisher’s exact test for categorical variables; Student’s *t* test for continuous variables with normal distribution and Mann–Whitney U test for continuous variables without a normal distribution.^5^ Economic class was defined by the ABEP (Brazil economic classification criterion) criterion. A or B: upper income status; C, D, or E: lower income status. ^6^ 1 US = R$ 3.44 at the time of data collection.

**Table 2:** Prevalence of anaemia and iron deficiency at baseline and after intervention.

Variable		MNP ^3^	FS_FA ^4^	*p* Value
Iron deficiency ^2^ (*n* = 108)	Baseline	72.09% (*n* = 54)	70.12% (*n* = 54)	0.821
	After intervention	39.17% (*n* = 28)	31.76% (*n* = 25)	0.417
	*p* value	<0.001	<0.001	
Anaemia ^1^ (*n* = 22)	Baseline	15.09% (*n* = 12)	11.89% (*n* = 10)	0.737
	After intervention	1.30% (*n* = 1)	1.34% (*n* = 1)	0.339
	*p* value	<0.001	<0.001	

The *p*-values were obtained by logistic regression, correcting for the effects of age and number of doses for each intervention. ^1^ Anaemia: hemoglobin measurement <11.0 g/dL. ^2^ Iron deficiency: soluble transferrin receptor >1.76 mg/L or serum ferritin <12 ng/mL (without inflammation) or serum ferritin >30 ng/mL (if C-reactive protein >0.5 mg/dL or alpha-1 acid glycoprotein >100 mg/dL). ^3^ MNP = Multiple micronutrients powder. ^4^ FS_FA = Ferrous sulfate and folic acid.

**Table 3:** Hemoglobin, serum ferritin, and soluble transferrin receptor means at baseline and after intervention.

Variables	Time	Baseline			After Intervention		
	Groups	MNP ^1^	FS_FA ^2^	*p* Value	MNP ^1^	FS_FA ^2^	*p* Value
Hemoglobin (g/dL) (ni = 162; nf = 151)	Global	11.68 ± 0.13 (*n =* 79)	11.64 ± 0.14 (*n* = 83)	0.909 ^3^	12.90 ± 0.11 (*n* = 75)	12.53 ± 0.12 (*n* = 76)	0.142 ^3^
	Anaemic	10.78 ± 0.24 (*n* = 12)	10.80 ± 0.27 (*n* = 10)	<0.001 ^4^	13.17 ± 0.23 (*n* = 11)	12.51 ± 0.24 (*n* = 10)	0.080 ^4^
	Non anaemic	12.58 ± 0.09 (*n* = 67)	12.48 ± 0.09 (*n* = 73)		12.62 ± 0.08 (*n* = 64)	12.54 ± 0.08 (*n* = 66)	
Serum ferritin (ng/mL) (ni = 161; nf = 152)	Global	25.97 ± 2.29 (*n* = 78)	26.13 ± 2.65 (*n* = 83)	0.378 ^3^	35.60 ± 2.33 (*n* = 76)	33.14 ± 2.48 (*n* = 76)	0.228 ^3^
	Anaemic	22.85 ± 4.29 (*n* = 12)	21.07 ± 5.11 (*n* = 10)	0.096 ^4^	38.90 ± 4.36 (*n* = 11)	29.36 ± 4.68 (*n* = 10)	0.112 ^4^
	Non anaemic	29.09 ± 1.77 (*n* = 66)	31.19 ± 1.67 (*n* = 73)		32.31 ± 1.76 (*n* = 65)	36.93 ± 1.71 (*n* = 66)	
Soluble transferrin receptor (mg/L) (ni = 161; nf = 150)	Global	2.00 ± 0.07 (*n* = 78)	2.18 ± 0.08 (*n* = 83)	0.294 ^3^	1.67 ± 0.04 (*n* = 74)	1.64 ± 0.04 (*n* = 76)	0.156 ^3^
	Anaemic	1.99 ± 0.13 (*n* = 12)	2.48 ± 0.15 (*n* = 10)	<0.001 ^4^	1.62 ± 0.07 (*n* = 10)	1.64 ± 0.08 (*n* = 10)	0.600 ^4^
	Non anaemic	2.01 ± 0.05 (*n* = 66)	1.87 ± 0.05 (*n* = 73)		1.71 ± 0.03 (*n* = 64)	1.64 ± 0.03 (*n* = 66)	

Values are expressed as the mean ± standard error of the mean. The means were obtained by adjusting the minimum squares. All analyses were adjusted for age. Serum ferritin was adjusted for C-reactive protein and alpha-1 acid glycoprotein. *n* = number of participants in the intervention group. ni = initial number of participants. nf = final number of participants. ^1^ MNP = Multiple micronutrients powder. ^2^ FS_FA = Ferrous sulfate and folic acid. ^3^ Significance of the difference between the effect of the two interventions at the baseline and after intervention—ANOVA. ^4^ Significance of the effect of groups within interventions (nested)—ANOVA. For serum ferritin, there was an effect of CRP (*p* < 0.001) and AGA (*p* = 0.012), which were used in all analyses as covariates.

**Table 4:** Delta values between periods (Δ: after intervention—baseline) ^1^.

Variables		MNP ^2^	FS_FA ^3^	*p* Value ^4^
Hemoglobin (g/dL) ^5^	Global	0.86 ± 0.17 (*n* = 75) d = 0.58	0.51 ± 0.18 (*n* = 76) d = 0.33	
	Anaemic	1.52 ± 0.25 (*n* = 11) d = 1.83	0.87 ± 0.27 (*n* = 10) d = 1.02	<0.001
	Non anaemic	0.19 ± 0.15 (*n* = 64) d = 0.16	0.17 ± 0.15 (*n* = 66) d = 0.14	
*p* value ^6^		0.382		
Serum ferritin (ng/mL) ^7^ (*n* = 143)	Global	7.84 ± 2.59 (*n* = 75) d = 0.35	3.52 ± 2.77 (*n* = 76) d = 0.15	
	Anaemic	12.01 ± 4.86 (*n* = 11) d = 0.75	−0.24 ± 5.22 (*n* = 10) d = −0.01	0.107
	Non anaemic	3.67 ± 1.96 (*n* = 64) d = 0.23	7.29 ± 1.91 (*n* = 66) d = 0.47	
*p* value ^6^		0.558		
Soluble transferrin receptor (mg/L) ^7^ (*n* = 141)	Global	−0.30 ± 0.06 (*n* = 73) d = −0.59	−0.44 ± 0.06 (*n* = 76) d = −0.84	
	Anaemic	−0.32 ± 0.12 (*n* = 10) d = −0.84	−0.65 ± 0.12 (*n* = 10) d = −1.71	0.011
	Non anaemic	−0.29 ± 0.05 (*n* = 63) d = −0.73	−0.24 ± 0.05 (*n* = 66) d = −0.59	
*p* value ^6^		0.962		

^1^ Values are expressed as mean and standard error of the mean. d = Cohen’s effect size. ^2^ MNP = Multiple micronutrients powder. ^3^ SF_SF = Ferrous sulfate and folic acid. ^4^ Significance of the difference between the effect of groups within interventions—ANOVA. ^5^ Complete mixed model. ^6^ Significance of the difference between the effect of interventions—ANOVA. ^7^ Fixed effects model.

## Data Availability

Data available on request due to restrictions, e.g., privacy or ethical. The data presented in this study are available on request from the corresponding author. The data are not publicly available due to additional analyses for future studies being produced based on these data.

## References

[1] Stevens G.A., Finucane M.M., De-Regil L.M., Paciorek C.J., Flaxman S.R., Branca F., Peña-Rosas J.P., Bhutta Z.A., Ezzati M., Nutrition Impact Model Study Group (2013). Behalf of Nutrition Impact Model Study Group Anaemia. Global, regional, and national trends in haemoglobin concentration and prevalence of total and severe anaemia in children and pregnant and non-pregnant women for 1995–2011: A systematic analysis of population-representative data. Lancet Glob. Health.

[2] De-Regil L.M., Jefferds M.E.D., Peña-Rosas J.P. (2017). Point-of-use fortification of foods with micronutrient powders containing iron in children of preschool and school-age. Cochrane Database Syst. Rev..

[3] World Health Organizaton World Health Data Platform. Prevalence of Anaemia in Children under 5 Years (%) [internet]. Geneva..

[4] Ministério da Saúde (Brasil) (2015). NutriSUS. Guia de evidências: Estratégia de Fortificação da Alimentação Infantil com Micronutrientes (Vitaminas e Minerais) em pó.

[5] World Health Organization (2014). Comprehensive Implementation Plan on Maternal, Infant, and Young Child Nutrition.

[6] De-Regil L.M., Suchdev P.S., Vist G.E., Walleser S., Peña-Rosas J. (2011). Home fortification of foods with multiple micronutrient powders for health and nutrition in children under two years of age. Cochrane Database Syst. Rev..

[7] Tam E., Keats E.C., Rind F., Das J.K., Bhutta Z.A. (2020). Micronutrient supplementation and fortification interventions on health and development outcomes among children under-five in low- and middle-income countries: A systematic review and meta-analysis. Nutrients.

[8] Suchdev P.S., Jefferds M.E.D., Ota E., Lopes K.S., De-Regil L.M. (2020). Home Fortification of Foods with Multiple Micronutrient Powders for Health and Nutrition in Children under Two Years of Age (Review).

[9] Christofides A., Asante K.P., Schauer C., Sharieff W., Owusu-Agyei S., Zlotkin S. (2006). Multi-micronutrient Sprinkles including a low dose of iron provided as microencapsulated ferrous fumarate improves haematologic indices in anaemic children: A randomized clinical trial. Matern. Child Nutr..

[10] Hirve S., Bhave S., Bavdekar A., Naik S., Pandit A., Schauer C., Christofides A., Hyder Z., Zlotkin S. (2007). Low dose ’Sprinkles’—An innovative approach to treat iron deficiency anemia in infants and young children. Indian Pediatr..

[11] Young M.F., Girard A.W., Mehta R., Srikantiah S., Gosdin L., Menon P., Ramakrishnan U., Martorell R., Avula R. (2018). Acceptability of multiple micronutrient powders and iron syrup in Bihar, India. Matern. Child Nutr..

[12] Inayati D.A., Scherbaum V., Purwestri R.C., Wirawan N.N., Suryantan J., Hartono S., Bloem M.A., Pangaribuan R.V., Biesalski H.K., Bellows A.C. (2012). Combined intensive nutrition education and micronutrient powder supplementation improved nutritional status of mildly wasted children on Nias Island, Indonesia. Asia Pac. J. Clin. Nutr..

[13] Kounnavong S., Sunahara T., Mascie-Taylor C.N., Hashizume M., Okumura J., Moji K., Boupha B., Yamamoto T. (2011). Effect of daily versus weekly home fortification with multiple micronutrient powder on haemoglobin concentration of young children in a rural area, Lao People’s Democratic Republic: A randomised trial. Nutr. J..

[14] Lundeen E., Schueth T., Toktobaev N., Zlotkin S., Hyder S.M.Z., Houser R. (2010). Daily use of Sprinkles micronutrient powder for 2 months reduces anemia among children 6 to 36 months of age in the Kyrgyz Republic: A cluster-randomized trial. Food Nutr. Bull..

[15] Macharia-Mutie C.W., Moretti D., Van den Briel N., Omusundi A.M., Mwangi A.M., Kok F.J., Zimmermann M.B., Brouwer I.D. (2012). Maize porridge enriched with a micronutrient powder containing low-dose iron as NaFeEDTA but not amaranth grain flour reduces anemia and iron deficiency in Kenyan preschool children. J. Nutr..

[16] Luo R., Yue A., Zhou H., Shi Y., Zhang L., Martorell R., Medina A., Rozelle S., Sylvia S. (2017). The effect of a micronutrient powder home fortification program on anemia and cognitive outcomes among young children in rural China: A cluster randomized trial. BMC Public Health.

[17] Somassè Y.E., Dramaix M., Traoré B., Ngabonziza I., Touré O., Konaté M., Diallo M., Donnen P. (2018). The WHO recommendation of home fortification of foods with multiple-micronutrient powders in children under 2 years of age and its effectiveness on anaemia and weight: A pragmatic cluster-randomized controlled trial. Public Health Nutr..

[18] Lanou H.B., Osendarp S.J.M., Argaw A., Polnay k Ouédraogo C., Kouanda S., Kolsteren P. (2019). Micronutrient powder supplements combined with nutrition education marginally improve growth amongst children aged 6–23 months in rural Burkina Faso: A cluster randomized controlled trial. Matern. Child Nutr..

[19] Larson L.M., Young M.F., Bauer P.J., Mehta R., Girard A.W., Ramakrishnan U., Verma P., Chaudhuri I., Srikantiah S., Martorell R. (2018). Effectiveness of a home fortification programme with multiple micronutrients on infant and young child development: A cluster-randomised trial in rural Bihar, India. Br. J. Nutr..

[20] Roschnik N., Diarra H., Dicko Y., Diarra S., Stanley I., Moestue H., McClean J., Verhoef H., Clarke S.E. (2019). Adherence and acceptability of community-based distribution of micronutrient powders in Southern Mali. Matern. Child Nutr..

[21] Ministério da Saúde (Brasil) (2015). NutriSUS. Manual Operacional: Estratégia de Fortificação da Alimentação Infantil com Micronutrientes (Vitaminas e Minerais) em pó.

[22] Cardoso M.A., Augusto R.A., Bortolini G.A., Oliveira C.S., Tietzman D.C., Sequeira L.A., Hadler M.C., do Rosario G., Peixoto M., Muniz P.T. (2016). Effect of providing multiple micronutrients in powder through primary healthcare on anemia in young brazilian children: A multicentre pragmatic controlled trial. PLoS ONE.

[23] Hadler M.C.C.M., Sigulem D.M., Alves M.F.C., Torres V.M. (2008). Treament and prevention of anemia with ferrous sulfate plus folic acid in children attending daycare centers in Goiânia, Goiás State, Brazil: A randomized controlled trial. Cad. Saúde Públic.

[24] HF-TAG—Home Fortification Technical Advisory Group (2013). Manual on Micronutrient Powder (MNPs) Composition.

[25] Associação Brasileira de Empresas de Pesquisas (ABEP) (2015). Critério de classificação econômica Brasil.

[26] World Health Organization (2011). Haemoglobin Concentrations for the Diagnosis of Anaemia and Assessment of Severity.

[27] Lundeen E.A., Lind J.N., Clarke K.E., Aburto N.J., Imanalieva C., Mamyrbaeva T., Ismailova A., Timmer A., Whitehead R.D., Serdula M.K. (2018). Four years after implementation of a national micronutrient powder program in Kyrgyzstan, prevalence of iron deficiency and iron deficiency anemia is lower, but prevalence of vitamin A deficiency is higher. Eur. J. Clin. Nutr..

[28] World Health Organization (2020). Guideline on Use of Ferritin Concentrations to Assess Iron Status in Individuals and Populations.

[29] Agência Nacional de Vigilância Sanitária—ANVISA (2002). Aprovação do Regulamento Técnico para a Fortificação das Farinhas de Trigo e das Farinhas de Milho com Ferro e Ácido Fólico.

[30] Ministério da Saúde (Brasil) (2000). Instituir o Componente I do Programa de Humanização no Pré-natal e Nascimento—Incentivo à Assistência Pré-natal no âmbito do Sistema Único de Saúde.

[31] Figueredo S.F., Mattar M.J.G., Abrao A.C.F.V. (2012). Iniciativa Hospital Amigo da Criança: Uma política de promoção, proteção e apoio ao aleitamento materno. Acta Paul. Enferm..

[32] Ministério da Saúde (Brasil) (2013). Programa Nacional de Suplementação de Ferro: Manual de condutas gerais.

[33] Ministério da Saúde (Brasil) (2013). Manual de condutas gerais do Programa Nacional de Suplementação de Vitamina A.

[34] Ministério da Saúde (Brasil) (2017). Bases para a discussão da Política Nacional de Promoção, Proteção e Apoio ao Aleitamento Materno.

[35] WHO—World Health Organization (2013). O clampeamento tardio do cordão umbilical reduz a anemia infantil.

[36] Giovannini M., Sala D., Usuelli M., Livio L., Francescato G., Braga M., Radaelli G., Riva E. (2006). Double-blind, placebo-controlled trial comparing effects of supplementation with two different combinations of micronutrients delivered as sprinkles on growth, anemia, and iron deficiency in cambodian infants. J. Pediatr. Gastroenterol. Nutr..

[37] Menon P., Ruel M.T., Loechl C.U., Arimond M., Habicht J.P., Pelto G., Michaud L. (2007). Micronutrient sprinkles reduce anemia among 9- to 24-mo-old children when delivered through an integrated health and nutrition program in rural Haiti. J. Nutr..

[38] Adu-Afarwuah S., Lartey A., Brown K.H., Zlotkin S., Briend A., Dewey K.G. (2007). Randomized comparison of 3 types of micronutrient supplements for home fortification of complementary foods in Ghana: Effects on growth and motor development. Am. J. Clin. Nutr..

[39] Hyder S.M.Z., Haseen F., Rahman M., Tondeur M., Zlotkin S.H. (2007). Effect of daily versus once-weekly home fortification with micronutrient sprinkles on hemoglobin and iron status among young children in rural Bangladesh. Food Nutr. Bull..

[40] Garcia-Casal M.N., Pasricha S.R., Sharma A.J., Peña-Rosas J.P. (2019). Use and interpretation of hemoglobin concentrations for assessing anemia status in individuals and populations: Results from a WHO technical meeting. Ann. N. Y. Acad. Sci..

[41] Kejo D., Petrucka P., Martin H., Mosha T.C.E., Kimanya M.E. (2019). Efficacy of Different Doses of Multiple Micronutrient Powder on Haemoglobin Concentration in Children Aged 6–59 Months in Arusha District. Scientifica.

[42] Carter R.C., Jacobson J.L., Burden M.J., Armony-Sivan R., Dodge N.C., Angelilli M.L., Lozoff B., Jacobson S.W. (2010). Iron deficiency anemia and cognitive function in infancy. Pediatrics.

[43] Tang M., Frank D.N., Hendricks A.E., Ir D., Esamai F., Liechty E., Hambidge K.M., Krebs N.F. (2017). Iron in micronutrient powder promotes an unfavorable gut microbiota in Kenyan infants. Nutrients.

